# Supplementary material for: Developing a Consensus-Based POCUS Protocol for Critically Ill Patients During Pandemics: A Modified Delphi Study
Source: Medicina (Kaunas). 2025 Jul 22;61(8):1319. doi: 10.3390/medicina61081319 (PMC12388432; doi:10.3390/medicina61081319)
Supplement: Supplementary file 1 [file medicina-61-01319-s001.zip › STable .pdf]

Supplemental Table S1. Result of the first Delphi survey.

| | | | | | | | | | |
|---|---|---|---|---|---|---|---|---|---|
| **Expert Delphi survey on items to be included in the point-of-care ultrasound (POCUS) protocol in the event of an infectious disaster** | | | | | | | | | |
| **Please indicate the degree of consent to the questions below in V [1=Very disagree~9=Very agree]** | | | | | | | | | |
| **POCUS-echocardiography** | | | | | | | | | |
| **Q1** | POCUS-echocardiography shall be included within this protocol. | | | | | | | | |
| **A1** | very disagree | | | | | | | | very agree |
| | 1 | 2 | 3 | 4 | 5 | 6 | 7 | 8 | 9 |
| | | | | | | 1/39 (3%) | 1/39 (3%) | 10/39 (26%) | 27/39 (69%) |
| **Q2** | Evaluate the left ventricle dysfunction newly discovered or aggravated by POCUS-echocardiography. | | | | | | | | |
| **A2** | very disagree | | | | | | | | very agree |
| | 1 | 2 | 3 | 4 | 5 | 6 | 7 | 8 | 9 |
| | | 1/39 (3%) | | | 1/39 (3%) | 1/39 (3%) | 1/39 (3%) | 12/39 (31%) | 23/39 (59%) |
| **Q3** | POCUS-echocardiography is used to assess whether there is a newly discovered RV dilatation or strain. | | | | | | | | |
| **A3** | very disagree | | | | | | | | very agree |
| | 1 | 2 | 3 | 4 | 5 | 6 | 7 | 8 | 9 |
| | | 1/39 (3%) | | | 1/39 (3%) | 2/39 (5%) | 4/39 (10%) | 12/39 (31%) | 19/39 (49%) |
| **Q4** | Evaluate the presence of large amounts of pericardial effusion and tamponade features with POCUS-echocardiography | | | | | | | | |
| **A4** | very disagree | | | | | | | | very agree |
| | 1 | 2 | 3 | 4 | 5 | 6 | 7 | 8 | 9 |
| | | | | | | | 5/39 (13%) | 7/39 (18%) | 27/39 (69%) |
| **Q5** | Evaluate hyperdynamic LV and IVC collapse with POCUS-echocardiography | | | | | | | | |
| **A5** | very disagree | | | | | | | | very agree |
| | 1 | 2 | 3 | 4 | 5 | 6 | 7 | 8 | 9 |
| | | | | 1/39 (3%) | 1/39 (3%) | 1/39 (3%) | 4/39 (10%) | 13/39 (33%) | 19/39 (49%) |
| **Q6** | If there is anything that needs to be added to the POCUS-echocardiography, please fill in (description) | | | | | | | | |
| **A6** | Evaluate the chamber size. Evaluate whether there is an AR. Determining if there is a flap in the Acknowledging aorta or not.<br>If it is included in the big term LV(RV) dysfunction/strain, it would be good to clarify the meaning.<br>I think we should evaluate that in itself. If 'new discovery' is applied, it may be necessary to add 'new' to finding in Lung US as well.<br>Grossed evaluation of valves (Severe stenosis - AS, MS, TS // Severe regurgitation - AR, MR, TR)<br>Need to add RWMA assessment<br>IVC plethora or IVC dilatation. RV dilatation or RV strain is acute or chronic in patients with shortness of breath.<br>detection of aneurysm | | | | | | | | |
| **POCUS-lung ultrasound** | | | | | | | | | |
| **Q7** | POCUS-lung ultrasound should be included within this protocol. | | | | | | | | |
| **A7** | very disagree | | | | | | | | very agree |
| | 1 | 2 | 3 | 4 | 5 | 6 | 7 | 8 | 9 |
| | | | | | | 3/39 (8%) | 4/39 (10%) | 5/39 (13%) | 27/39 (69%) |
| **Q8** | POCUS-lung ultrasound scans six areas of the chest (see attachment 2) | | | | | | | | |
| **A8** | very disagree | | | | | | | | very agree |
| | 1 | 2 | 3 | 4 | 5 | 6 | 7 | 8 | 9 |
| | | 1/39 | | | | 3/39 (8%) | 10/39 (26%) | 14/39 (365) | 11/39 (28%) |
| **Q9** | Check if A-line and Lung sliding are present with POCUS-lung ultrasound. | | | | | | | | |
| **A9** | very disagree | | | | | | | | very agree |
| | 1 | 2 | 3 | 4 | 5 | 6 | 7 | 8 | 9 |
| | | | | | | 1/39 (3%) | 6/39 (15%) | 7/39 (18%) | 25/39 (64%) |
| **Q10** | The presence, distribution, and density of B-line are evaluated with POCUS-lung ultrasound. | | | | | | | | |
| **A10** | very disagree | | | | | | | | very agree |
| | 1 | 2 | 3 | 4 | 5 | 6 | 7 | 8 | 9 |
| | | | | | 1/39 (3%) | 4/39 (10%) | 5/39 (13%) | 9/39 (23%) | 20/39 (51%) |
| **Q11** | The presence or absence of pleural effusion is evaluated by POCUS-lung ultrasound. | | | | | | | | |
| **A11** | very disagree | | | | | | | | very agree |
| | 1 | 2 | 3 | 4 | 5 | 6 | 7 | 8 | 9 |
| | | | | | 1/39 (3%) | 1/39 (3%) | 4/39 (10%) | 11/39 (28%) | 22/39 (56%) |
| **Q12** | The presence or absence of consolidation is evaluated by POCUS-lung ultrasound. | | | | | | | | |
| **A12** | very disagree | | | | | | | | very agree |
| | 1 | 2 | 3 | 4 | 5 | 6 | 7 | 8 | 9 |
| | 1/39 (3%) | | 1/39 (3%) | 1/39 (3%) | 3/39 (8%) | 5/39 (13%) | 8/39 (21%) | 8/39 (21%) | 12/39 (31%) |
| **Q13** | The presence or absence of pneumothorax is evaluated by POCUS-lung ultrasound | | | | | | | | |
| **A13** | very disagree | | | | | | | | very agree |
| | 1 | 2 | 3 | 4 | 5 | 6 | 7 | 8 | 9 |
| | | | | | 1/39 (3%) | 2/39 (5%) | 8/39 (21%) | 10/39 (26%) | 18/39 (46%) |
| **Q14** | The condition of lung is evaluated by synthesizing findings such as B-line, lung sliding, pleural irregularities, pleural effect, and consolidation | | | | | | | | |
| **A14** | very disagree | | | | | | | | very agree |
| | 1 | 2 | 3 | 4 | 5 | 6 | 7 | 8 | 9 |
| | | | | 1/39 (3%) | 1/39 (3%) | 2/39 (5%) | 6/39 (15%) | 8/39 (21%) | 21/39 (54%) |
| **Q15** | If there is anything that needs to be added to the POCUS-echocardiography, please fill in (description) | | | | | | | | |
| **A15** | It may take too much time to conduct a comprehensive exam to evaluate the presence or absence of consolidation with pulmonary ultrasound in adults.<br>I think it is possible to check the existence and location of the line, and it may be difficult to check the density.<br>You can miss the post area's pneumonia to zone 6.<br>Comprehensive LUS may be required to increase DDx sensitivi | | | | | | | | |
| **Other POCUS ultrasound or additional imaging tests** | | | | | | | | | |
| **Q16** | Both lungs are A-line with newly developed RV dilatation or RV stain, and if there is a lung sliding, suspect pulmonary embolism and consider DVT US or lung CT. | | | | | | | | |
| **A16** | very disagree | | | | | | | | very agree |
| | 1 | 2 | 3 | 4 | 5 | 6 | 7 | 8 | 9 |
| | | | | 1/39 (3%) | | 2/39 (5%) | 7/39 (18%) | 9/39 23%) | 20/39 (51%) |
| **Q17** | If the cardiac echocardiogram exhibits hyperdynamic LV, the IVC is collocated, and there is A-line and lung sliding in the field pulmonary echocardiogram | | | | | | | | |

| | | | | | | | | | |
|---|---|---|---|---|---|---|---|---|---|
| | consider FAST and aortic ultrasound and abdominal CT | | | | | | | | |
| **A17** | very disagree | | | | | | | | very agree |
| | 1 | 2 | 3 | 4 | 5 | 6 | 7 | 8 | 9 |
| | 1/39 (3%) | 1/39 (3%) | | | 1/39 (3%) | | 4/39 (10%) | 12/39 (31%) | 20/39 (51%) |
| **Q18** | If you have any additional comments regarding POCUS, please write them down. (description) | | | | | | | | |
| **A18** | It seems necessary to distinguish between disease/trauma.<br>This protocol seems to be limited to emergency medicine specialists who are enhanced expertise.<br>It is necessary to distinguish between pulmonary embolism and RV infraction among the causes of acute RV failure.<br>It is considered difficult to apply it in consideration of the privacy of the patient (especially female patients).<br>If PTE is suspected in POCUS, DVT US is an option and should proceed directly to lung CT.<br>Pulmonary hypertension should also be considered. | | | | | | | | |

Supplemental Table 2. Result of the second Delphi survey.

| | | | | | | | | | |
|---|---|---|---|---|---|---|---|---|---|
| **Expert Delphi survey on items to be included in the point-of-care ultrasound (POCUS) protocol in the event of an infectious disaster** | | | | | | | | | |
| **Please indicate the degree of consent to the questions below in V [1=Very disagree~9=Very agree]** | | | | | | | | | |
| **OCUS-echocardiography** | | | | | | | | | |
| **Q1** | Consider ACS w/u if you show RWMA in case of new or aggravated left ventricle dysfunction or RV dilatation or strain with POCUS-echocardiography | | | | | | | | |
| **A1** | very disagree | | | | | | | | very agree |
| | 1 | 2 | 3 | 4 | 5 | 6 | 7 | 8 | 9 |
| | | | | | **4/39 (10%)** | **1/39 (3%)** | **7/39 (18%)** | **14/39 (36%)** | **13/39 (33%)** |
| **Q2** | If POCUS-echocardiography is normal and A-line + long sliding is seen in LUS, but the patient's dyspnea is continued, auscultation is performed to check COPD, asthma, etc., and additional tests are considered if necessary to confirm other metabolic causes. | | | | | | | | |
| **A2** | very disagree | | | | | | | | very agree |
| | 1 | 2 | 3 | 4 | 5 | 6 | 7 | 8 | 9 |
| | | **1/39 (3%)** | | | **3/39 (8%)** | **3/39 (8%)** | **6/39 (16%)** | **12/39 (31%)** | **14/39 (36%)** |
| **Q3** | If there is anything that needs to be added to POCUS-echocardiography, please fill it out (description) | | | | | | | | |
| **A3** | Set the criteria for field judgment for LV dysfunction, RV dilatation, or strain so that ultrasonic waves can be performed within a limited time and resources.<br>RWMA can occur without appearing with LV or RV dysfunction.<br>It would be better to start with the stethoscope.<br>If there are abnormal findings or clinical symptoms, additional tests are naturally necessary.<br>If RWMA is suspected without Lt. ventral dysfunction or RV dilatation/strain, it is questionable whether it would be appropriate to consider infection first.<br>Chronic RV strain is highly likely with COPD. POCUS-echocardiography may not be normal.<br>EPSS for evaluation of left ventricular systolic function and TAPSE for evaluation of right ventricular systolic function by measuring the mitral valve flow.<br>PE as well as ACS should be considered. Stress-induced CMP is also possible, and heart and inferior venous ultrasound must be done first. | | | | | | | | |
| **POCUS-lung ultrasound** | | | | | | | | | |
| **Q4** | POCUS-lung ultrasound scans 6 areas of the chest, but if 6 areas are normal but pneumonia is strongly suspected, consider adding the back side. | | | | | | | | |
| **A4** | very disagree | | | | | | | | very agree |
| | 1 | 2 | 3 | 4 | 5 | 6 | 7 | 8 | 9 |
| | **1/39 (3%)** | | **3/39 (8%)** | **3/39 (8%)** | **5/39 (13%)** | | **13/39 (33%)** | **9/39 (23%)** | **5/39 (13%)** |
| **Q5** | If there is anything that needs to be added to the POCUS-lung ultrasound, please write it down (description) | | | | | | | | |
| **A6** | In actual clinical practice, pneumonia or fluid collection is often present only in the dependent positioning.<br>It is difficult to diagnose pneumonia only with on-site ultrasound.<br>I think it will be necessary in situations such as ICU setting where it is not easy to perform radiologic examination.<br>Iif CT is available, it is likely that CT will be considered before additional ultrasound in question<br>I think it's right to do a comprehensive LUS<br><br>For most Dyspnea patients, it is thought that it will be covered in six areas, and after that, it will be efficient to conduct blood tests and consider CT if possible. | | | | | | | | |
| | **Other POCUS or additional imaging tests** | | | | | | | | |
| **Q6** | If you have RV dilatation or strain with POCUS-echocardiography, but you see a B-line in LUS, there is no lung sliding, consider additional w/u | | | | | | | | |
| **A6** | very disagree | | | | | | | | very agree |
| | 1 | 2 | 3 | 4 | 5 | 6 | 7 | 8 | 9 |
| | | | | **1/39 (3%)** | **6/39 (16%)** | **7/39 (18%)** | **8/39 (21%)** | **9/39 (23%)** | **8/39 (21%)** |
| **Q7** | Aorta CT is considered if aortic detection is suspected if there is a large amount of pericardial effect and tamponade feature with POCUS-echocardiography | | | | | | | | |
| **A7** | very disagree | | | | | | | | very agree |
| | 1 | 2 | 3 | 4 | 5 | 6 | 7 | 8 | 9 |
| | **1/39 (3%)** | | **2/39 (5%)** | **1/39 (3%)** | **2/39 (5%)** | **2/39 (5%)** | **5/39 (13%)** | **10/39 (26%)** | **16/39 (41%)** |
| **Q8** | If you have any additional comments regarding POCUS, please write them down. (description) | | | | | | | | |
| **A8** | There should be a standard for judging tamponade quickly by on-site ultrasound.<br>The protocol between pericardial tamponade and aortic detection seems to be limited. Consider chest CT for evaluation of aortic dissection.<br>In case of suspected aortic detection, ultrasound is performed first through supra-sternal notch view or paramagnetic short axis and CT is considered.<br>ARDS is likely in this case. ARDS, septic CMP, which can cause acute corpulmonale, should also be considered.<br>Consider pericardiocentesis, if table, cardiology cosnult and cause w/u.<br><br>Consider DVT US and/or advanced imaging, and anti-coagulation start when PTE is suspected. | | | | | | | | |
